# Mechanisms Underlying Cancer Progression Caused by Ezrin Overexpression in Tongue Squamous Cell Carcinoma

**DOI:** 10.1371/journal.pone.0054881

**Published:** 2013-01-24

**Authors:** Shota Saito, Hiroto Yamamoto, Ken-ichi Mukaisho, Sho Sato, Tomoki Higo, Takanori Hattori, Gaku Yamamoto, Hiroyuki Sugihara

**Affiliations:** 1 Department of Pathology, Division of Molecular Diagnostic Pathology, Shiga University of Medical Science, Otsu, Shiga, Japan; 2 Department of Oral and Maxillofacial surgery, Shiga University of Medical Science, Otsu, Shiga, Japan; Vanderbilt University Medical Center, United States of America

## Abstract

**Background:**

Ezrin is a member of the ezrin, radixin, and moesin family that provides a functional link between the plasma membrane and the cortical actin cytoskeleton. A correlation between ezrin overexpression and aggressive cancer behavior has been recently reported in various tumor types. However, its roles in the mechanisms underlying progression of tongue squamous cell carcinoma (SCC) are unclear.

**Method:**

We used human tongue SCC and noncancerous tissue microarrays to immunohistochemically analyze the ezrin expression level and its relationship with proliferative activity. The human tongue SCC cell line HSC-3 was used to determine the effects of ezrin RNA interference (RNAi) on cancer cells during MTT; wound healing and invasion assays; immunofluorescence of the actin cytoskeleton; and western blotting of E-cadherin, N-cadherin, β-catenin, and the active and total RhoA/Rac1/cdc42.

**Results:**

Ezrin was overexpressed in 46.4% of the tumors examined in human tongue SCC tissue microarrays. Ezrin expression was correlated with the Ki-67 index. Ezrin depletion by RNAi in the HSC-3 cells significantly reduced cell proliferation, migration, and invasiveness and disturbed actin reorganization during podia formation. Its effects on RhoA/Rac1/cdc42 expression were not significant, whereas it enhanced E-cadherin and β-catenin expression and decreased N-cadherin expression.

**Conclusions:**

Ezrin is often overexpressed in primary tongue SCCs and may have an important role in their growth, migration, and invasiveness possibly via its relationship with the E-cadherin/β-catenin complex and the cadherin switch. Thus, ezrin could be a therapeutic target in tongue SCC.

## Introduction

Head and neck squamous cell carcinoma (HNSCC) is currently the seventh most common cancer, with 260,000 new cases diagnosed each year and approximately 128,000 annual deaths worldwide [1], [2]. The tongue is one of the most common sites of origin for HNSCC in Japan [3]. Neck lymph node metastasis is one of the most critical determinants of survival and provides a good guide for treatment strategies [4]–[8]. However, the quality of life and the five-year survival rate are low in advanced tongue cancers even with current multimodal therapy and surgical excision accompanied by chemotherapy and radiotherapy. To improve the outcomes of advanced tongue cancers, we need to develop new targeted therapies based on an understanding of the molecular mechanisms underlying the aggressive behavior of tongue cancers.

Ezrin was initially isolated as a cytoskeletal component of intestinal microvilli, and it is known to be a substrate of tyrosine kinase [9]. Ezrin is a member of the ezrin, radixin, and moesin protein family that links F-actin to cell membrane proteins after phosphorylation [10]–[13]. This linker function suggests that ezrin is essential for many fundamental cellular processes, including determination of the cell shape, polarity, surface structure, cell adhesion, motility, cytokinesis, phagocytosis, and integration of membrane transport through signaling pathways [14]–[17]. These functional aspects of ezrin are expected to promote tumor progression. Indeed, recent studies have revealed that ezrin may have an important role in tumorigenesis, development, invasion, and metastasis, probably through regulation of adhesion molecules, participation in cell signal transduction, and signaling to other cell membrane channels in the tumor [18]–[22]. Ezrin is an indispensable factor for tumor cell metastasis in osteosarcomas [23], breast cancer [24], nasopharyngeal carcinomas [25], and prostatic cancer [26]. Ezrin expression has also been linked to poor survival in several cancers, including carcinomas of the breast [21], [27], endometrium [28], and ovary [29]; cutaneous and uveal melanomas [30]; and soft tissue sarcomas [31], [32]. However, its roles in oral cancer are unclear. This study aimed to clarify the roles of ezrin in tongue SCC progression with ezrin RNA interference (RNAi) in a cell line derived from tongue SCC. We used primary tongue SCCs to determine the frequency of ezrin overexpression and the correlations of ezrin expression with the Ki-67 index and the apoptotic index, which reflect contributions of cell proliferation and cell loss, respectively, to tumor growth and aggressiveness. Our results suggest that ezrin may be suitable for targeted gene therapy in tongue SCCs.

## Materials and Methods

### Immunohistochemical staining of ezrin and histological examination

The normal and tumor tongue tissue microarrays of humans used in this study were obtained from US Biomax Company (MD, USA). Of the 79 samples, 10 were normal tongue tissues and 69 were tongue SCC tissues. US Biomax Company obtained the tissue resources from tissue banks who guaranteed that all human tissue collections were performed at certified hospitals according to the highest ethical standards. All human tissues were also collected according to protocols that complied with the Health Insurance Portability and Accountability Act (HIPPA). They certified that all tissue banks who provided human tissue resources met the following requirements of the Human Material Transfer Agreement: the donor’s identity was anonymized and all tissues and data were labeled using an ID-code to protect the identity of the tissue donors. Informed consent was kept at tissue banks and not provided to US Biomax Company, thereby protecting the donor’s privacy.

Immunohistochemical staining of the human tongue SCC tissue microarrays was performed using an anti-ezrin rabbit antibody (#3145; Cell Signaling, MA, USA) and a monoclonal mouse anti-Ki-67 antibody (clone: MIB-1; Dako, Glostrup, Denmark). Staining was performed using a Discovery XT Automated IHC Stainer with a Ventana DABMap Detection Kit (No. 760–124; Ventana Medical System, AZ, USA). Each step of the Ventana DABMap Detection Kit procedure was optimized on the Discovery XT instrument, and the conditions were preset. Antigen retrieval from the tissue sections was performed using heat.

The staining intensity of human tongue SCC tissue was graded as follows: 0, negative; 1+, weak; 2+, moderate; and 3+, intense. This grading used the following criteria: 1+ indicated an intensity similar to that found in the normal tongue tissue; 3+ indicated intense staining of the membrane and cytoplasm; 2+ indicated an intensity between 1+ and 3+. We dichotomized the categories during statistical analysis. Thus, a weak–moderate staining intensity indicated low ezrin expression, whereas an intense staining intensity indicated high ezrin expression.

Ki-67 is usually expressed in the cell nucleus. The Ki-67 index (i.e., the number of Ki-67-positive tumor cells divided by the total number of tumor cell × 100%) was determined by counting the number of tumor cells in three randomly selected high-power fields (×400).

The apoptotic index was measured using an In situ Apoptosis Detection Kit (Takara Bio, Otsu, Japan). The staining procedures followed the manufacturer’s instructions. After routine deparaffinization, the tissue was digested with proteinaseK (20 µg/mL in PBS) for 15 min at room temperature and washed with PBS. Slides were then incubated in 3% hydrogen peroxide for 5 min and washed with PBS. TdT enzyme and substrate was pipetted onto the sections, which were then incubated at 37°C for 90 min. After washing, anti-FITC HRP conjugate was added to the slides for 30 min. The slides were washed, stained with diaminobenzine (Nichirei, Tokyo, Japan), and counterstained with hematoxylin. The apoptotic index (the number of positive tumor cells divided by the total number of tumor cells × 100%) was determined by counting the number of tumor cells in three randomly selected high-power fields (×400). The correlations between ezrin expression, Ki-67, and the apoptotic index were also evaluated in human tongue SCC tissues.

### Cell culture

The tongue SCC cell line HSC-3 was purchased from the JCRB Cell Bank and maintained in Dulbecco’s modified Eagle’s medium (DMEM; Nacalai Tesque, Kyoto, Japan) containing 10% fetal bovine serum (FBS) and 1% antibiotic–antimycotic solution (Invitrogen, CA, USA) at 37°C in a humidified atmosphere supplemented with 5% CO_2_.

### Small interfering RNA (siRNA)

Ezrin siRNA (5′-GAUUUCCUACCUGGCUGAAGCUGGA-3′) and nonsilencing control (NSC) siRNA were purchased from Invitrogen. Cells were transfected using Lipofectamine RNAiMAX reagent (Invitrogen), according to the manufacturer’s instructions. The ezrin siRNA-transfected HSC-3 cells (ezrin-siRNA) and NSC-transfected HSC-3 cells (NSC) were used in all of the *in vitro* experiments.

### Quantitative reverse transcription-polymerase chain reaction (qRT-PCR)

In the qRT-PCR, the total RNA was isolated from cell lines using RNeasy (Qiagen, Tokyo, Japan) and cDNA was synthesized from 2 µg of the total RNA. cDNA was subjected to PCR (LightCycler480, Roche, Tokyo, Japan) using primers and SYBR Premix Ex Taq II (Takara Bio). All PCR primers were purchased from Takara Bio and their sequences are shown in Table 1. PCR was performed using the following conditions: denaturing at 95°C for 30 s, followed by 40 cycles of PCR at 95°C for 5 s, annealing at 60°C for 20 s, and elongation at 72°C for 10 s. The mRNA expression levels were normalized against the mRNA expression levels of the internal standard gene glyceraldehyde-3-phosphate dehydrogenase (GAPDH).

**Table 1:** Primer Sequences used for qRT-PCR.

Ezrin	Forward	5′- ACCATGGATGCAGAGCTGGAG -3′
	Reverse	5′- ACATAGTGGAGGCCAAAGTACCACA -3′
GAPDH	Forward	5′-GCACCGTCAAGGCTGAGAAC-3′
	Reverse	5′-TGGTGAAGACGCCAGTGGA-3′

### Western blotting

Confluent cells were lysed in lysis buffer (50 mM Tris–HCl, pH 7.4, 150 mM sodium chloride, 0.5 mM EDTA, 0.09 units/mL aprotinin, 1 µg/mL pepstatin, 10 mM phenylmethylsulfonyl fluoride, and 1 µg/mL leupeptin). Protein lysates (20 µg per lane) detected in the BCA protein assay were separated using a 4%–12% SDS-PAGE gradient gel (NuPAGE, Invitrogen) and transferred onto polyvinylidene difluoride membranes (Invitrogen). Membranes were blocked with 4% nonfat dried milk in TBS-T (20 mM Tris–HCl, pH 7.5, 8 g/L sodium chloride, and 0.1% Tween 20) before incubating with primary antibodies.

Anti-ezrin rabbit antibody (#3145) was purchased from Cell Signaling Technology. Anti-β-actin mouse monoclonal antibody (sc-47778) and anti-N-cadherin mouse antibody (sc-7939) were purchased from Santa Cruz Biotechnology (CA, USA). Anti-E-cadherin mouse antibody (clone 36) and anti-β-catenin mouse antibody (clone 14) were purchased from Becton Dickinson and Company (BD; NJ, USA). Goat peroxidase-conjugated anti-rabbit IgG (#L3012; Signalway Antibody, TX, USA) and goat peroxidase-conjugated anti-mouse IgG (#AP124P; Millipore, MA, USA) were used as secondary antibodies. β-actin was used as the internal positive control. Proteins were visualized using HRP substrate (Millipore) and scanned with an enhanced chemiluminescence system (Las 4000; Fuji Film, Tokyo, Japan). The band intensities were normalized to β-actin.

### Cell cycle and apoptosis assay

Cells (1×10^5^) were seeded into 75-cm^2^ flasks and transfected with ezrin or negative control siRNA. Cells were harvested three days after transfection and prepared for flow cytometric analysis. Cells were fixed for 30 min in 70% ethanol and stained with propidium iodide. Measurements of the DNA cellular content were performed using a FACSCalibur (BD).

### Cell growth assay

An MTT assay was used to evaluate cell growth. Cells were seeded in 12-well plates (1×10^4^ cells/well), and the MTT solution (0.25 mg/mL) was added to the medium after incubation for 24, 48, 72, or 96 h. Formazan crystals were dissolved in DMSO, and absorption was measured at 570 nm using an Infinite 200 microplate reader (TECAN, Kawasaki, Japan).

### Cell migration assay

Migration was evaluated using a wound healing assay. Cells were grown in 12-well plates to a near-confluent level in DMEM containing 10% FBS. Crossed streaks were made on the monolayer culture using 200-µL pipette tips. The cells were washed immediately with DMEM containing 10% FBS to remove the detached cells. Cells were incubated in a medium containing mitomycin (3 µg/mL; Nacalai Tesque) for 1 h to inhibit proliferation. Thus, the observed increase in cell number was not attributable to increased cellular proliferation. Cell migration was monitored for 0, 12, 24, and 48 h, and images were captured at each time point using a digital camera (Nikon, Tokyo, Japan) attached to an inverted phase contrast microscope (Nikon).

### Invasion assay

The invasion assays used 24-well BD BioCoat Matrigel invasion chambers with 8-µm pore inserts (BD). Cells (5×10^4^) suspended in serum-free DMEM were seeded in the upper inserts, while DMEM containing 10% FBS was added to the lower chambers as a chemoattractant. The cells were removed from the upper surface of the filter by scraping with a cotton swab after 22 h in culture. Cells that infiltrated through the filter were fixed and stained with hematoxylin–eosin (H/E). The mean values of the results obtained with the three chambers were used in the analysis.

### Immunofluorescence

Cells grown in eight-well culture slides were fixed with 3.7% formaldehyde in PBS for 30 min, permeabilized with 0.25% Triton X-100 (Merck CalBiochem, Darmstadt, Germany), and blocked with 1% FBS in PBS. Actin was stained using rhodamine–phalloidin (Invitrogen). Immunofluorescence images were visualized using an Olympus BX-61 fluorescent microscope (Olympus, Tokyo, Japan), and images were captured with a CoolSNAP-HQ camera (NIPPON ROPER, Tokyo, Japan).

### Rho family activity assay

Rho family activation assay was also performed using an RhoA/Rac1/Cdc42 Activation Combo Kit (Cell Biolabs, San Diego, CA, USA), according to the manufacturer’s instructions. In brief, cells were washed, lysed in assay buffer, and centrifuged at 10,000×*g* for 1 min to remove cell debris. To determine the total expression levels of RhoA/Rac1/Cdc42 by western blotting, 20 µL of each sample was stored at −80°C for separate analysis. A cell lysate containing 500 µg of total protein was incubated for 1 h at 4°C with rotation with 40 µL of the agarose beads, which bound activated RhoA, Rac1, and Cdc42. Agarose beads bound to active RhoA/Rac1/Cdc42 were washed three times using assay buffer, resuspended in 40 µL of 2× LDS sample buffer, and boiled at 70°C for 10 min. The active (GTP-bound) and total RhoA/Rac1/Cdc42 protein levels in each sample were determined by western blotting.

### Statistical analyses

Each experiment was performed in triplicate. All data were expressed as the mean ± SD. Statistical analysis was performed using Excel (Microsoft, WA, USA). Comparisons between groups were conducted using the Student’s *t*-test. The correlation of the ezrin expression levels with tongue SCC and lymph node metastasis was analyzed using Fisher’s exact test, whereas the stage and differentiation grades were analyzed using Tukey’s multiple comparison method. Differences were considered statistically significant at *P*<0.05.

## Results

### Ezrin expression in tongue cancer and noncancerous tissues

**Figure 1:**
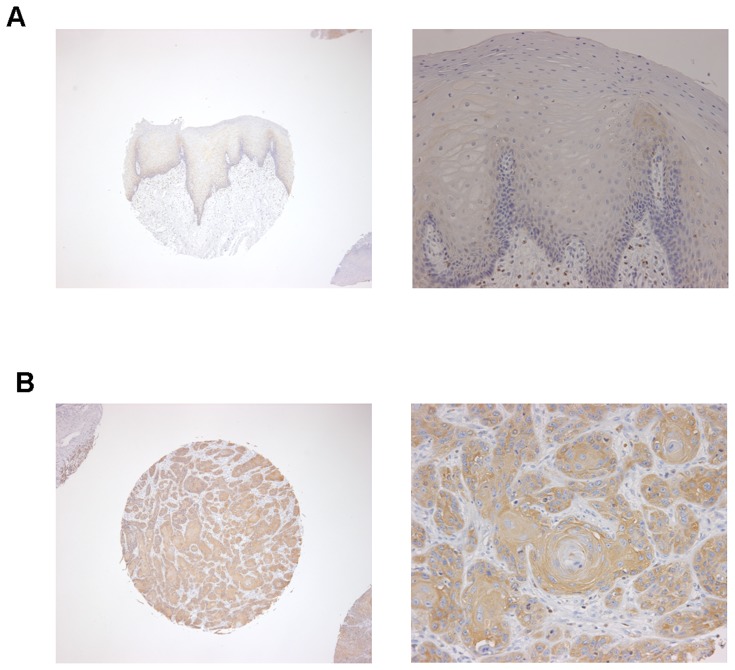
Immunohistochemical staining of ezrin in human tongue SCCs and noncancerous tissues. (A) Ezrin expression was low in noncancerous tissues (magnification 40× and 200×). (B) Ezrin was highly expressed in tongue squamous cell carcinomas (magnification 40× and 200×). Ezrin immunoreactivity was apparent in the membrane of normal tongue epithelium, whereas positive staining for ezrin was primarily in the cytoplasm or in the membrane and cytoplasm of tongue squamous cell carcinoma cells.

Ezrin immunoreactivity was observed in the cell membranes, while it was weakly positive in the cytoplasm of normal tongue mucosa (Fig. 1A). In contrast, tongue SCC samples demonstrated membranous and cytoplasmic ezrin staining, and the cytoplasmic staining was greater in these samples than in the normal tongue mucosa samples (Fig. 1B). Table 2 presents the results of ezrin staining. In noncancerous human tongue tissues, all 10 samples expressed ezrin at low levels. In human tongue SCC tissues, however, high ezrin expression was detected in 32/69 (46.4%) tumors, whereas low expression was detected in 37/69 (53.6%) tumors. High ezrin expression was significantly more common in human tongue SCC tissues than in noncancerous tissues (*P* = 0.0046). High ezrin expression tended to be detected more frequently in the cases with lymph node metastasis (62.5%) than in with those without lymph node metastasis (44.3%), but there was no significant correlation between the ezrin expression levels and the stage grade, regional lymph node metastasis, and the differentiation grade (*P*>0.05).

**Table 2:** Association between ezrin expression and clinico-pathologic variables in 79 tongue tissue.

		Ezrin expression		
		low	high	
Normal	n = 10	10 (100%)	0 (0%)	
				*P = 0.0046*
Tongue squamous cell carcinoma	n = 69	37 (56.3%)	32 (46.4%)	
TNM staging				
stage I	n = 24	12 (50.0%)	12 (50.0%)	
stage II	n = 29	18 (62.1%)	11 (37.9%)	
stage III	n = 12	5 (41.7%)	7 (58.3%)	
stage IV	n = 4	2 (50.0%)	2 (50.0%)	
Lymph node metastasis				
lymph node metastasis−	n = 61	34 (55.7%)	27 (44.3%)	
lymph node metastasis+	n = 8	3 (37.5%)	5 (62.5%)	
Pathology diagnosis				
Grade1(well-differentiated)	n = 50	26 (52.0%)	24 (48.0%)	
Grade2(moderately-differentiated)	n = 11	6 (54.5%)	5 (45.5%)	
Grade3(poorly-differentiated)	n = 8	5 (62.5%)	3 (37.5%)	
Grade4(undifferentiated)	n = 0	–	–	

### Correlations between ezrin expression and the indices of Ki-67 and apoptosis in human tongue SCC tissues

**Figure 2:**
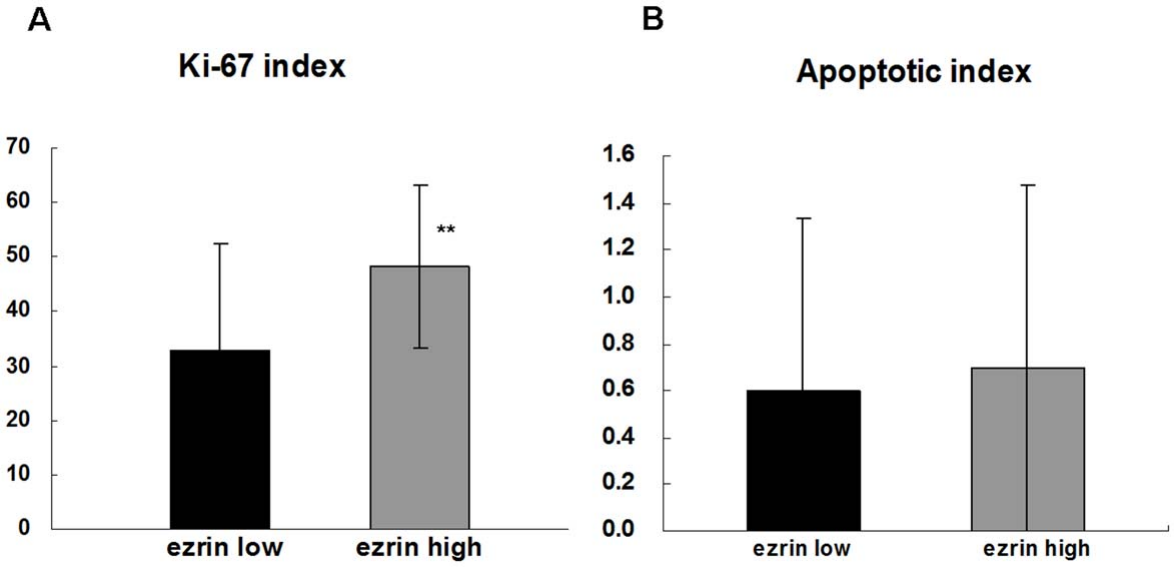
Correlations between ezrin expression and the indices of Ki-67 and apoptosis in human tongue SCC tissues. (A) The Ki-67 index was 33.0±19.3% in tissues with low ezrin expression and 48.1±15.0% in tissues with high ezrin expression. Significant differences were observed in correlation between ezrin expression and the Ki-67 index (*P* = 0.0003). (B) There was no significant correlation between ezrin expression and apoptotic indices (*P* = 0.5776).

We evaluated the correlations between ezrin expression and the Ki-67 and apoptotic indices in human tongue SCC tissues. The Ki-67 index was 33.0±19.3% in tissues with low ezrin expression levels and 48.1±15.0% in tissues with high ezrin expression levels (Fig. 2A). There was a positive correlation between ezrin expression levels and the Ki-67 index (*P* = 0.0003). The apoptotic index was 0.60±0.74% in tissues with low ezrin expression levels and 0.70±0.78% in tissues with high ezrin expression levels (Fig. 2B). There was no significant correlation between the ezrin expression levels and the apoptotic index (*P* = 0.5776).

### Ezrin gene expression in the human tongue SCC cell line HSC-3 after RNAi

**Figure 3:**
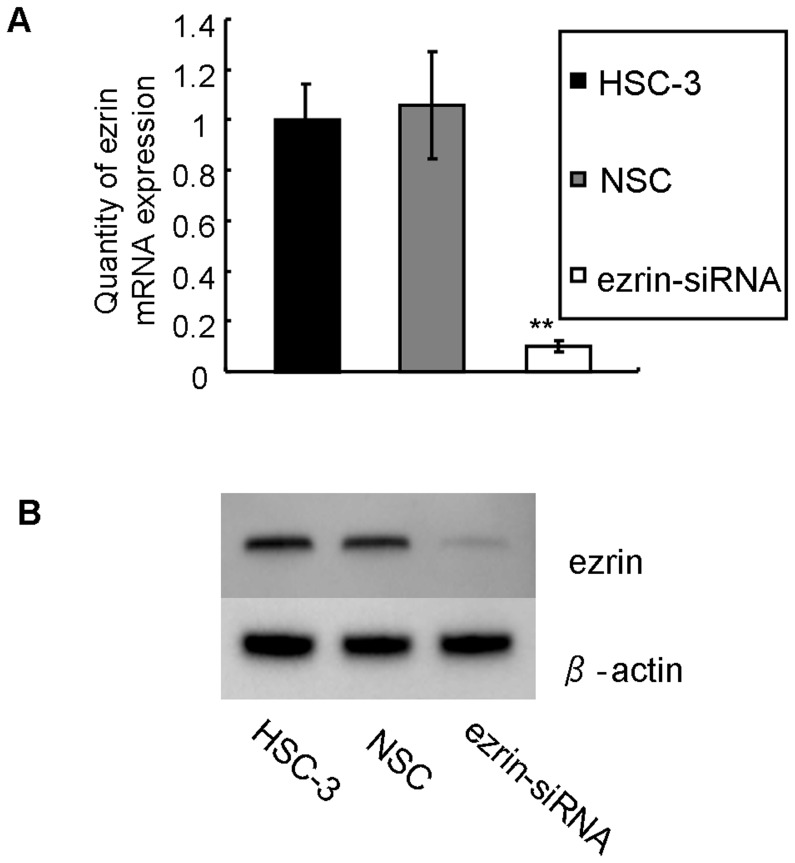
The expression of ezrin in HSC-3 tongue SCC cells after siRNA treatment. (A) Ezrin mRNA expression was analyzed using real-time PCR after RNAi. The inhibition of ezrin mRNA expression was clearly observed in ezrin siRNA-transfected HSC-3 cells compared with that in HSC-3 control cells (0.10±0.03 vs 1.06±0.21; *P*<0.05). (B) Ezrin protein expression was detected by western blotting after RNAi. The expression of ezrin decreased dramatically in ezrin siRNA-transfected HSC-3 cells.

To examine the effects of ezrin siRNA treatment on the HSC-3 cells, we used qRT-PCR and western blotting to measure the ezrin mRNA and protein levels, respectively, in the HSC-3 cells that were transfected with siRNA. As shown in Figure 3A, the ezrin mRNA expression was clearly inhibited in the ezrin-siRNA cells than in the NSC cells (0.10±0.03 vs 1.06±0.21; *P*<0.05). The western blot analysis demonstrated that RNAi reduced the ezrin protein levels (Fig. 3B).

### Effects of ezrin RNAi on cell cycle progression and apoptosis

**Figure 4:**
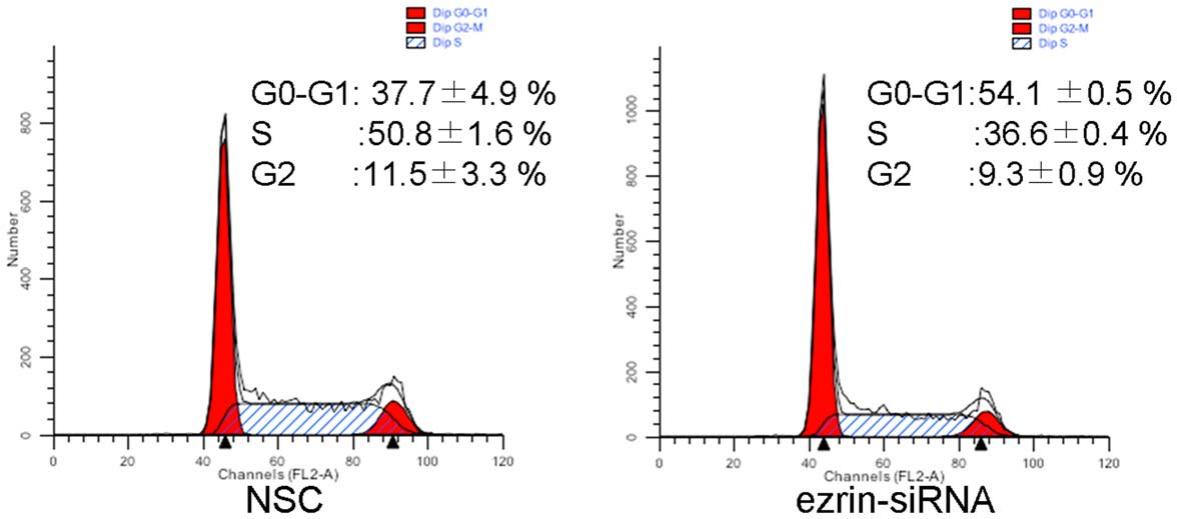
Cell cycle changes after RNAi treatment. The proportion of HSC-3 cells in the S phase decreased from 50.8±1.6% to 36.6±0.4% after RNAi (*P* = 0.0018). The proportion of cells in the G0/G1 phase also increased from 37.7±4.9% to 54.1±0.5% after RNAi (*P* = 0.0018). Apoptosis was not detected.

We transfected the HSC-3 cells with ezrin siRNA and collected them after three days for cell cycle analysis. Flow cytometry showed that the G0/G1 fraction increased from 37.7%±4.9% to 54.1%±0.5% (*P* = 0.0046) in the HSC-3 cells after RNAi (Fig. 4). In contrast, the S and G2/M fractions were decreased in the HSC-3 cells after RNAi. In particular, the proportions of S phase cells decreased from 50.8%±1.6% to 36.6%±0.4% (*P* = 0.0018), whereas the proportion of the G2/M cells decreased from 11.5%±3.3% to 9.3%±0.9% (*P* = 0.1155). No preG0/G1 cells or apoptotic bodies were observed in the control and siRNA-transfected cells. These results suggest that ezrin siRNA may inhibit cell proliferation by interfering with cell mitosis and cell cycle progression.

### Effects of ezrin RNAi on cell growth

**Figure 5:**
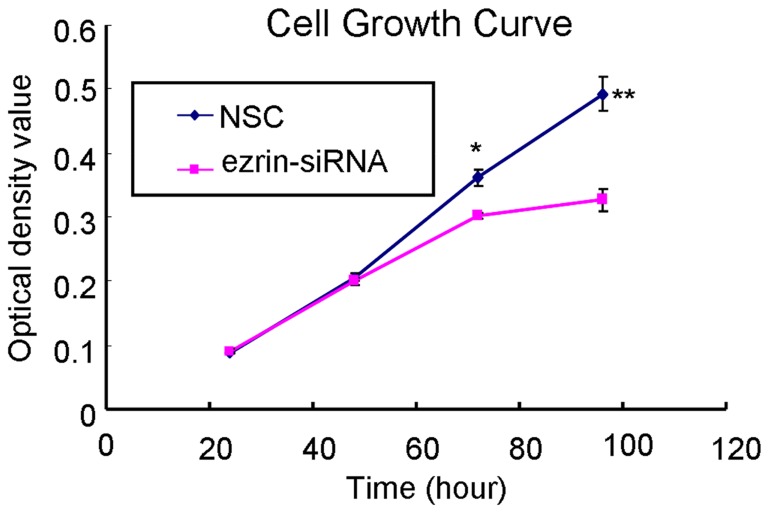
Effects of ezrin downregulation on HSC-3 cell growth. The growth of ezrin siRNA-transfected HSC-3 cells decreased in a time-dependent manner. (24 h: *P* = 0.6727; 48 h: *P* = 0.5314; 72 h: *P* = 0.0122; 96 h: *P* = 0.0065).

The effects of ezrin protein expression on cell growth were tested using an MTT assay. The growth rate of the ezrin siRNA-treated cells decreased in a time-dependent manner (Fig. 5). The absorbance values of the MTT assay after 72 h were 0.36±0.02 and 0.30±0.01 for the NSC- and ezrin siRNA-transfected HSC-3 cells, respectively. These data indicate that there was a significant reduction in cell growth in the ezrin siRNA-transfected cells than in the control cells.

### Effects of ezrin RNAi on cell migration and invasion

**Figure 6:**
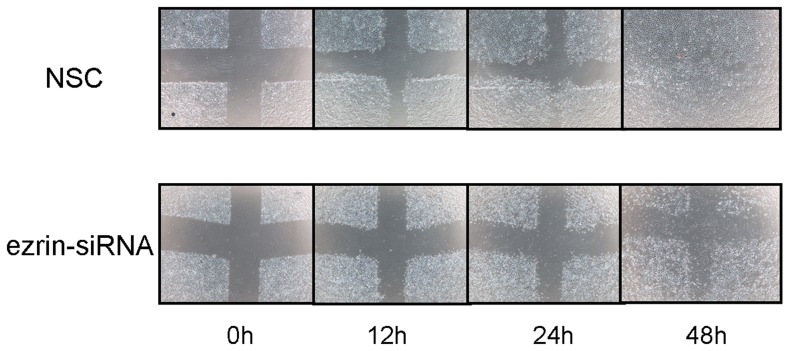
Cell wound healing assay. Cells were wounded by scratching with a pipette tip, and mitomycin (3 mg/mL) was added to the medium for 1 h to inhibit the proliferation of cancer cells. Cells were subsequently incubated with DMEM for 48 h. Cells were photographed using phase-contrast microscopy. HSC-3 cell migration was reduced by ezrin siRNA treatment.

**Figure 7:**
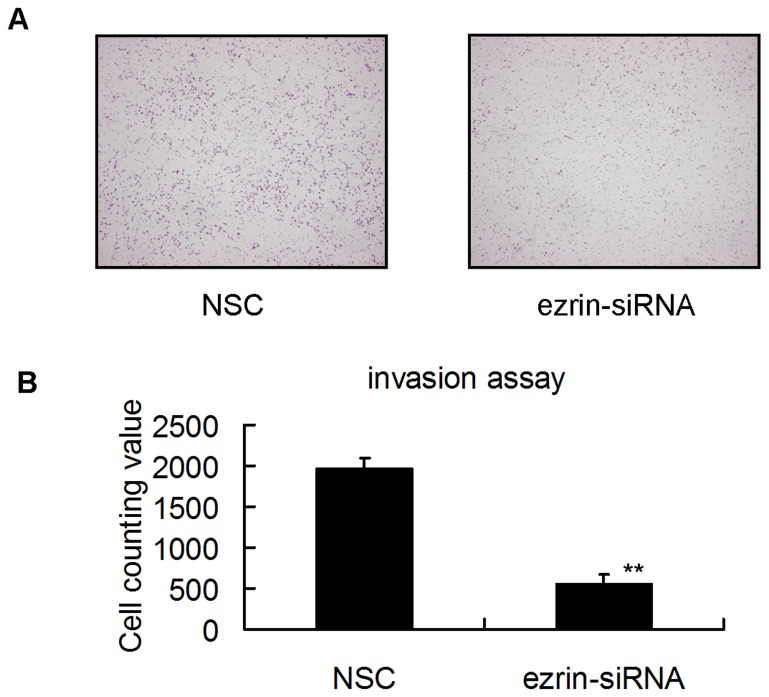
Invasion assay. (A) Matrigel-coated Transwell chambers were used to analyze cell invasion. Cells that infiltrated through the filter were fixed and stained, and representative fields were photographed. (B) The cells were quantified by counting under a light microscope. Compared with the HSC-3 control cells, the ezrin siRNA-transfected HSC-3 cells exhibited significantly decreased invasiveness (1969.8±126.6 vs 564.7±111.8; *P*<0.05).

Wound healing assays were conducted to evaluate the cell motility of the NSC- and ezrin siRNA-transfected cells. A wound was created on a cell monolayer, and wound closure was assessed at various time points. Ezrin silencing in the HSC-3 cells impaired their ability to heal a wound compared with that in control cells that expressed ezrin (Fig. 6). Invasion assays were also performed using Matrigel-coated Transwell culture chambers, and the invading cells were counted after 22 h. The ezrin siRNA-transfected cells exhibited a four-fold lower cell invasion rate than the NSC-transfected cells that expressed ezrin (564.7±111.8 vs 1969.8±126.6; *P*<0.05; Fig. 7).

### Effects of ezrin RNAi on actin cytoskeleton reorganization

**Figure 8:**
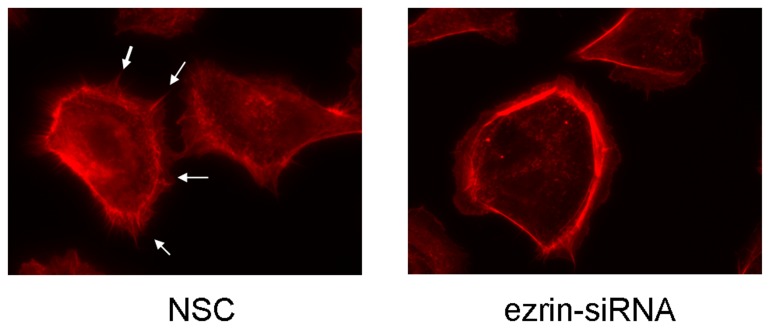
Immunofluorescence. NSC- and ezrin siRNA-transfected HSC-3 cells were labeled with an antibody against actin. Ezrin depletion of HSC-3 cells led to reduced ezrin protein levels. This downregulation was associated with the loss of protrusions (arrows).

We analyzed the effects of ezrin downregulation on the organization of the actin cytoskeleton. Analyses of phalloidin-stained cells showed that podia formation was clearly inhibited in the ezrin siRNA-transfected cells (Fig. 8). These findings indicated that the absence of ezrin caused morphological changes in cancer cells through actin cytoskeleton remodeling.

### Rho family proteins, E-cadherin, N-cadherin, and β-catenin expression in the ezrin-depleted cells

**Figure 9:**
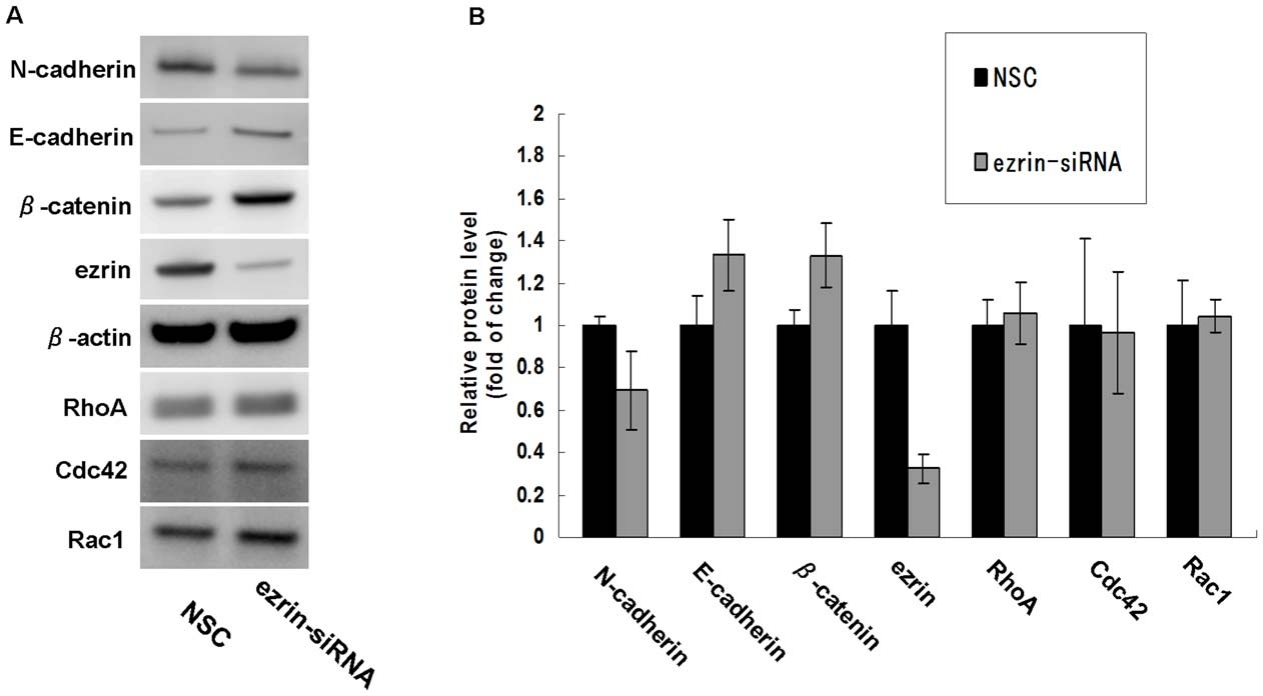
Western blot analyses of E-cadherin, N-cadherin, β-catenin, and Rho family proteins in HSC-3 cells. The expression of E-cadherin and β-catenin was increased and the expression of N-cadherin was decreased in ezrin siRNA-transfected HSC-3 cells compared with that in the NSC-transfected cells. There was no significant difference in the total protein levels of RhoA, Rac1 and Cdc42.

There were no differences in the total protein levels of RhoA, Rac1, and Cdc42 (Fig. 9), and the levels of their active forms were too low to be detected in the NSC- and ezrin siRNA-transfected cells.

We tested whether the expression levels of E-cadherin, N-cadherin, and β-catenin were affected by ezrin depletion in the HSC-3 cells by quantifying their expression using western blotting. As shown in Figure 9, E-cadherin and β-catenin expressions were increased, whereas N-cadherin expression was decreased in the ezrin siRNA-transfected cells than in the NSC-transfected cells. These results indicate that suppression of ezrin protein expression induced the upregulation of E-cadherin and β-catenin but the downregulation of N-cadherin in the HSC-3 cells.

## Discussion

Tissue microarray analyses showed that high ezrin expression was significantly more common in the human tongue SCC tissues than the noncancerous tissues (*P* = 0.0046). There was also a positive correlation between ezrin expression and the Ki-67 index in this study. The Ki-67 antigen is expressed by proliferating cells during the G1, S, G2, and M phases, but not during the G0 phase (resting cells) [33]. Ki-67 is used as a marker of tumor proliferation and aggressiveness, and it can have a major effect on the prognosis of patients with HNSCC [34]. High ezrin expression tended to be detected more frequently in cases with lymph node metastasis (62.5%) than in those without lymph node metastasis (44.3%). Our *in vitro* experiments using the human tongue SCC cell line HSC-3 with and without RNAi treatment also detected an association between ezrin overexpression and more aggressive behavior, whereas there were no significant correlations between the ezrin expression patterns and TNM staging in human tongue SCC tissues. Nicolas et al. also reported no significant difference in ezrin expression in advanced and lower TNM stage tumors, whereas high levels of ezrin and moesin expression were associated with poor cancer survival [35], [36]. These findings suggest that ezrin overexpression can be used as a prognostic marker independently of TNM staging.

The *in vitro* roles of ezrin in cellular behavior have not been evaluated in tongue SCCs. Our RNAi experiments detected strong suppression of cell growth, motility, and invasiveness in the HSC-3 tongue SCC cells. The cell cycle analysis revealed that ezrin depletion increased the G0/G1 fraction but decreased the G2-M fraction by interfering with cell mitosis and cell cycle progression. These results agree with those reported by Zhang et al. in hepatocellular carcinoma [37]. They also indicated that ezrin could enhance the growth of cancer cells by supporting cell division and cell cycle progression from G0/G1 to the S and G2/M phases. This agreed with the results of the tissue microarray Ki-67 index. We also observed that ezrin depletion inhibited cell growth in an MTT assay.

Cell migration and invasion are essential processes in the metastasis of cancer cells. Migratory cancer cells undergo a series of morphological changes via reorganization of adhesion molecules and actin fibers [38]. It is now widely accepted that the process of cancer cell migration involves four main steps—formation and extension of filopodia and lamellipodia at the leading edge, establishment of new adhesion sites at the front, contraction of the cell body, and detachment of adhesions at the rear [39]. We observed that ezrin depletion decreased cancer cell motility and invasiveness and inhibited podia formation. These results indicate that ezrin inhibition disturbed remodeling of the actin cytoskeleton, which reduced the motility and invasiveness of the HSC-3 cells. These findings agree with previous reports, which showed that changes in the cytoskeleton may be a key factor in the regulation of neoplastic progression and tumor growth [24], [40]–[43]. A previous study also detected a dramatic reduction in the number of pancreatic cancer cells with podosomal rosettes after ezrin RNAi treatment and reported that formation of podosomes and their rosettes was driven by an ezrin–cortactin interaction, which has a role in pancreatic cancer invasion [44]. Another report revealed that pseudopod formation was clearly decreased by ezrin RNAi treatment in four hepatocellular carcinoma cell lines [37].

Our results suggest that ezrin plays an important role in the invasive growth of tongue SCCs. We also examined actin, Rho family proteins, E-cadherin, N-cadherin, and β-catenin to investigate the molecular mechanisms underlying these observations. We found that ezrin suppression induced the enhanced expression of E-cadherin and β-catenin in the HSC-3 cells. It is known that E-cadherin plays a central role in epithelial cell–cell adhesion and the maintenance of epithelial cell colony integrity [45]. It is well documented that the loss or inhibition of E-cadherin function, or its associated catenins, leads to reduced intercellular adhesion and an increased invasive potential [46]. Moreover, several studies of epithelial malignancies, including oral SCCs, have suggested that the E-cadherin/β-catenin complex regulates cancer invasion and metastasis [47], [48]. Cadherin adhesion complexes interact with the actin cytoskeleton in a complex manner, which is poorly understood. The current view is that E-cadherin is linked to the actin cytoskeleton through its interaction with β-catenin, which in turn binds the actin-binding protein α-catenin [49]. Recent evidence suggests that ezrin is important for localization of E-cadherin to the plasma membrane [24]. Thus, we hypothesized that the E-cadherin and β-catenin downregulation linked to ezrin overexpression may have been associated with actin remodeling and the formation of podia extensions.

It is widely known that actin reorganization is regulated by the Rho family small GTPases such as Rho, Rac, and Cdc42 [50], i.e., Rho regulates stress fiber and focal adhesion assembly, Rac regulates the formation of lamellipodia protrusions and membrane ruffles, and Cdc42 triggers filopodial extensions at the cell periphery [51]. We examined the expression and activity of RhoA, Rac1, and Cdc42 in the ezrin siRNA- and NSC-transfected HSC-3 cells. However, there were no differences in the total protein levels of RhoA, Rac1, and Cdc42, and the level of their active forms were too low to detect, irrespective of ezrin-siRNA transfection. It is unclear whether these findings reflect the low significance of Rho family proteins in the motility of the HSC-3 cells.

In our RNAi experiments, ezrin suppression induced E-cadherin upregulation and N-cadherin downregulation. This indicates that ezrin may be correlated with cadherin switching and epithelial-to-mesenchymal transitions (EMT). Numerous studies have suggested that EMT is a potent mechanism that enhances the detachment of cancer cells from a primary tumor and their migration into the tumor stroma, vessels, and metastatic sites [52]–[54]. Cadherin switching is also essential for increased motility [54]. Previous reports indicate that ezrin is associated with several signaling cascades. In colon cancer, ezrin interacts with L1CAM and regulates NF-κB signaling [55]. Ezrin silencing downregulates the MAPK and TGF-β pathways in esophageal SCC [56]. Ezrin also participates in the activation of MAPK and PI3K in breast and prostate cancer [57]. There is a possibility that ezrin affects cadherin switching and EMT induction through signaling modifications. Further studies are necessary to explore the correlation between ezrin expression and EMT.

Ezrin is known to be essential for many fundamental cellular processes [14]–[17] and is also related to the malignant behavior of various malignant tumors [18]–[32]. The present study suggests that ezrin is also related to the malignant behavior of tongue SCC cells through various aspects of the functional roles of ezrin, including its upregulation of cell growth by accelerating cell cycle progression and its upregulation of cell motility and invasiveness through remodeling of actin fibers and podia formation. Ezrin was often overexpressed in primary tongue SCCs; thus, it may also play an important *in vivo* role in the malignant behavior of tongue SCCs. This suggests that ezrin may be a potential target for the treatment of human tongue cancers.
